# Awareness and care seeking for long COVID symptoms among Coronavirus disease survivors in Bahir Dar City, Northwest Ethiopia: phenomenological study

**DOI:** 10.1186/s12889-023-15889-0

**Published:** 2023-05-24

**Authors:** Kassawmar Angaw Bogale, Taye Zeru, Molalign Tarkegn, Melashu Balew, Masetewal Worku, Anemaw Asrat, Ayinengida Adamu, Yared Mulu, Atalay Getachew, Fentie Ambaw

**Affiliations:** 1 School of Public Health, College of Medicine and Health Sciences, Bahir Dar University, Bahir Dar, Ethiopia; 2 Amhara Public Health Institute, Bahir Dar, Ethiopia; 3 Rennes University, Rennes, France; 4 Department of Environmental Health, College of Health Sciences, Debre Markos University, Debre Markos, Ethiopia

**Keywords:** Post-Acute COVID-19 Syndrome, Awareness, Health Care Seeking Behavior

## Abstract

**Background:**

Corona Virus Disease (COVID-19) has long-term sequels that persisted for months to years and manifested with a spectrum of signs and symptoms. Presentations of long COVID-19 symptoms are heterogeneous, vary from person to person, and can reach up to over 200 symptoms. Limited studies are conducted on the awareness of long COVID-19. So, this study aimed to explore the awareness about and care seeking for long COVID-19 symptoms among COVID survivors in Bahir Dar City in 2022.

**Methods:**

A qualitative study with a phenomenological design was used. Participants of the study were individuals who survived five months or longer after they tested positive for COVID-19 in Bahir Dar city. Individuals were selected purposively. An in-depth interview guide was prepared and used to collect the data. Open Cod 4.03 software was used for coding and synthesizing. Thematic analysis was used to analyze the transcripts.

**Results:**

The themes emerged from the data were awareness, experience of symptoms and their effects, and care practices of long COVID-19. Although only one participant mentioned the common symptoms of long COVID-19 the survivors experienced general, respiratory, cardiac, digestive, neurological, and other symptoms. These symptoms include rash, fatigue fever, cough, palpitations, shortness of breath, chest pain, and abdominal pain, loss of concentration, loss of smell, sleep disorder, depression, joint and muscle pain. These symptoms brought various physical and psychosocial effects. The majority of the respondents described that long COVID-19 symptoms will go off by themselves. To alleviate the problems some of the participants had taken different measures including medical care, homemade remedies, spiritual solutions, and lifestyle modification.

**Conclusions:**

The result of this study revealed that participants have a significant deficit of awareness about the common symptoms, risk groups, and communicability of Long COVID. However, they experienced the majority of the common symptoms of Long COVID. To alleviate the problems, they had taken different measures including medical care, homemade remedies, spiritual solutions, and lifestyle modification.

## Introduction

Corona Virus Disease (COVID-19) has long-term sequels that persisted months to years and manifested with a spectrum of signs and symptoms among people who have a history of either confirmed or probable severe acute respiratory syndrome coronavirus 2 (SARS-CoV-2) infections. The condition was first identified, and named as long COVID, by a patient-led group of researchers [1] ‘in Spring 2020 to describe their journeys of not recovering’[2]. In addition to the term, Long COVID different names have been suggested in the literature including post-COVID-19 conditions, post-acute sequelae of SARS-CoV-2 infection [1], post-acute COVID-19 syndrome, post-acute COVID, chronic COVID, long-haul COVID [3].

Presentations are heterogeneous, vary from person to person, and can reach up to over 200 symptoms. The symptoms can be new onsets, recurrent or persistent ones [4]. Multiple organ systems are affected by the Long COVID including the brain, the lung and the heart, and the gastrointestinal system[5]. Common symptoms of Long COVID include shortness of breath, fatigue, memory impediments or generally cognitive dysfunction[4, 6, 7], gastrointestinal symptoms [6], insomnia, cough, depression, change or loss of smell/taste, atypical chest pain [5], headache, loss of focus, and hair loss [7]. Generally, illnesses are considered to have an impact on everyday functioning[4].

The time for the onset and stay of the symptoms of Long COVID ranges from months to years and as time goes on symptoms decline. Given that the manifestations are heterogeneous and that the time of onset and duration of stay of Long COVID cases vary from individual to individual. Compounded with the lack of awareness, especially among the community, about the pathogenesis, long-term effects, and syndrome, there is no consensus on the case definition of Long COVID [8].

The subset of COVID patients that lead to Long COVID can be 20% to 30% of those who were mild cases of SARS-CoV-2 infection or asymptomatic and nearly 50% of those who have been admitted and discharged [6]. Risk factors for the persistence of symptoms among non-hospitalized adult patients include being female, being older age, belonging to an ethnic minority, being deprived socioeconomically, smoking, being obese, and having a wide range of comorbidities [9].

Different qualitative studies have provided valuable insight into the lived experience of long COVID-19 [10, 11]. People with long COVID describe an illness trajectory and heterogeneous symptomatology that did not conform to initial expectations and were not acknowledged in public health advice [10, 11]. This has posed challenges for the medical profession, who have lacked evidence-based guidance to treat and support patients. Individual experiences of medical care have varied from well-meaning but inconclusive to disbelief [10, 11]. This lack of understanding about long COVID extends beyond the medical and scientific communities to employers, family, and friends, leaving many people feeling frustrated and isolated in their self-management efforts and increasingly turning to online communities of people with long COVID for validation and advice [10, 11].

Community awareness about COVID-19 transmission, signs/symptoms, treatment, and prevention strategies have a great impact on the dynamicity of the disease in distribution, severity, and staying long across the globe [12, 13] including our setting. Experiences indicate an urgent need to better understand the individual experience of long-COVID and help clinicians understand what is needed to help such patients in their recovery.

The finding of this study will have incredible benefits for the community, the health professionals, the programmer, and the policymakers, as well as for researchers. The community will be benefited from appreciating their level of awareness and health-seeking behavior at the time of long COVID-19. This might also be a great opportunity for the community to increase the level of understanding of disease prevention and rapid recovery action. So, this study aimed to explore the awareness about and care seeking for long COVID-19 symptoms among COVID survivors in Bahir Dar City in 2022.

## Methods

### Study area and period

Bahir Dar is the capital city of Amhara National Regional State of Ethiopia with a variety of attractions in the nearby Lake Tana and Blue Nile River. It is one of the ten most beautiful cities in Africa and one of the twelve UNESCO learning city awardees of 2015 [14]. The city is located 564 km Northwest of Addis Ababa. In 2022, it had a total population of 422,580, of whom 207,064 were male. About 81% of the population is urban dwellers. There are three public hospitals, 10 health centers, 15 health posts, 16 private clinics, and 4 private general hospitals. The study was conducted from September to October 2022. The first case of COVID-19 in Amhara region was detected on 30 March 2020 in Bahir Dar city. As of 14 August 2022, 16,583 confirmed cases of COVID-19 (4528 RDT confirmed) were detected regionally. 497 COVID-19-related deaths (3.00% CFR) were recorded and 16,055 patients (s) recovered from COVID-19 (96.8% recovery rate). Of which 5, 129 confirmed cases, 1,316 patients recovered and 166 COVID-19-related deaths were reported in Bahir Dar city [15].

As part of the response to the pandemic, isolation of the suspected individuals in Bahir Dar City was made in Bahir Dar Military general hospital, Felege Hiwot, and Tibebe-Ghion Comprehensive Specialized hospitals. The diagnostic services were given at the Amhara Public Health Institute and Tibebe-Ghion specialized hospital. Currently, the testing service is also started in private health facilities.

The vaccination coverage of COVID-19 in Bahir Dar city was 10.8% of AstraZeneca vaccine/ AZD1222_1st Dose and 8.4% of AZD1222_2nd Dose. Hence, the vaccination performance of the city is very low [16].

## Study design

We used a phenomenological study design to explore the awareness about and care seeking for long COVID-19 symptoms among COVID survivors in Bahir Dar City.

### Participants of the study

Participants in the study were people over the age of eighteen who had survived for five months or more after testing positive for COVID-19 in the city of Bahir Dar.

### Sample size

Three major perspectives; sex, hospitalization history, and age were considered. The final sample size was determined by the saturation of ideas during data collection and analysis. Four participants were interviewed from each perspective.

### Sampling techniques and recruitment

Individuals who survived COVID-19 were selected purposively with an intensity-sampling technique to explore their awareness as well as their caring experiences of long COVID.

COVID-19 survivors who tested positive for five months or longer were recruited from the admission logbook in the Tibebe Ghion specialized hospital. In addition, we used snowball technique to identify study participants.

Eligible participants were reached through phone calls to confirm their willingness to participate in the study to arrange for an in-depth interview at nearby health facilities.

### Data collection tool and procedures

In-depth interviews were conducted to get information on the awareness about and care seeking of long COVID symptoms. Nine researchers (PhD students) performed the interviews. All responders provided their informed consent. A structured interview guide that was written in Amharic (the local language) was used to conduct the interviews. Before beginning the actual data collecting, the interview guide was pre-tested. Two days of training for data collectors were given in order to harmonize the data collection process. Face to face interviews were conducted to assess the participants' feelings. The entire interview has been conducted under COVID 19 precautions.

### Data analysis

Individual interviews were transcribed word by word, and contextual translation was made. Open Cod 4.03 software was used for coding and synthesizing. Thematic Analysis was used to analyze the transcripts. To ensure inter-rater reliability, all the investigators analyzed data, and interpretations of themes were shared and discussed to agree upon key themes that emerged from the data.

Close and repeated reading of transcripts, regular discussion about emerging findings, and triangulation of findings were done to strengthen the quality of the analysis.

### Trustworthiness

Credibility: To assure the credibility of the response, participants with a higher probability of experiencing long COVID-19 symptoms were selected and they were asked to talk about their own experiences regarding the symptoms and their severity in more detail than general. During data collection, the consistency of the participants' responses was also checked through frequent probing.

#### Dependability

The study was undertaken by PhD students with different educational backgrounds. During data collection, since there was a mix in the understanding of the question, the researchers agreed to include a statement with a clear definition about long COVID-19 at the beginning of the question. During data analysis, the translated document of the 23 participants was first coded from P1-P23. The responses for each question were collected from 23 participants and compiled in a word document. Coding was done by displaying the compiled word document for all the researchers.

#### Conformability

To assure the conformability of the study, contextual translations were done by individual researchers and this was rechecked by research team members. The interpretation of the findings was derived from the data.

### Ethical consideration

Ethics clearance was obtained from the ethical review board of the College of Medicine and Health Sciences, Bahir Dar University. A support letter was obtained from the Amhara Public Health Institute. Written informed consent was obtained from study participants. Participants were told the study's purpose, risks, and benefits and requested to put their signature on the informed consent form. Fortunately, all participants were able to read and write. Participants were assured that all data was de-identified, stored, and handled anonymously. The privacy of the study participants and confidentiality of the data was maintained.

## Results

### Socio-demographic characteristics of respondents

From a total of 23 selected study participants, 10 (43.5%) were females. Most of the study respondents, 19 (82.6%), had an educational level of diploma and above. Only three (13%) of the participants were vaccinated (Table 1).

**Table 1:** Socio-demographic characteristics of participants in Bahir Dar town, Ethiopia, 2022 (*n* = 23)

Variables	Frequency	Percent (%)
**Sex**		
Male	13	56.5
Female	10	43.5
**Age category**		
< 65	20	87.0
≥ 65	3	13.0
**Educational level**		
No formal education	1	4.3
Grade 1–8	2	8.7
Grade 9–12	1	4.3
Diploma and above	19	82.6
**Occupation**		
Government Employer	8	34.8
Private	6	26.0
NGO	3	13.0
Unemployed	5	21.7
**Clinical History**		
Hospitalized	17	73.9
Non hospitalized	6	16.1
**Comorbidity**		
Yes	10	43.5
No	13	56.5
**Prior vaccination**		
Vaccinated	3	13.0
Not vaccinated	20	87.0

### Awareness about and care seeking for long COVID-19 symptoms

**Fig. 1:**
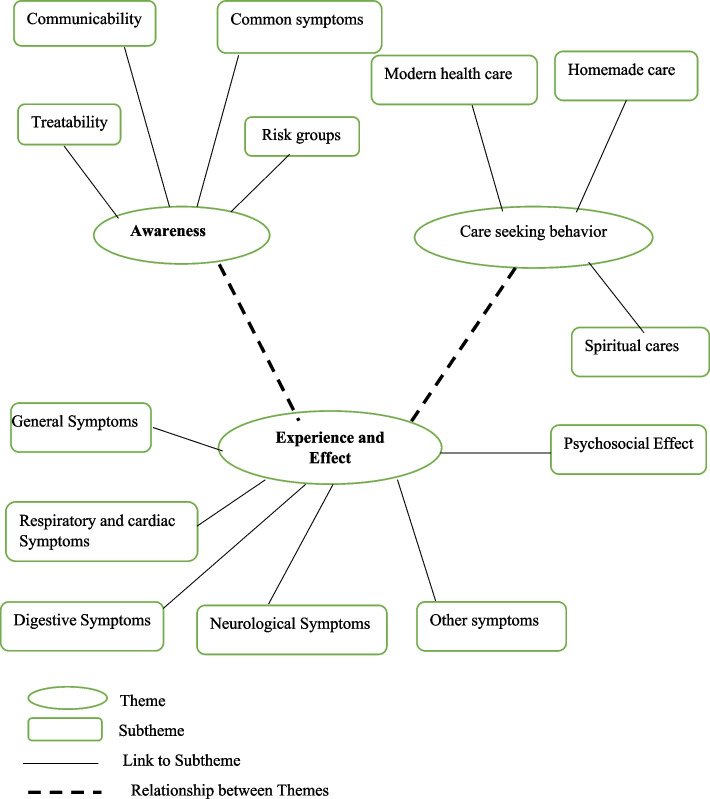
Thematic map indicating three themes

The 23 interviews produced over 108 pages of transcripts and notes. Study participants reported symptoms, risk groups, communicability, experience, care advice, and practice of long COVID-19. During the analysis, 83 codes, 12 sub-themes, and three themes were generated. The themes were awareness, experience of symptoms and their effects, and care practices of long COVID-19 (Fig. 1). Themes and selected quotations are organized as follows.

### Theme I: Awareness about long COVID 19

Most of the participants did not mention the 19 common symptoms. None of the participants described palpitations, headache, pin or needle feeling, loss of smell, diarrhea, stomach pain, rash, and change in the menstrual cycle. In addition, they associate some of these symptoms with non-long COVID-19 conditions like Asthma, diabetes, and hypertension.

*"I do not know the symptom of long COVID. But, I face difficulty when I want to breathe. I was treated for Asthma the last time but it was not Asthma.* [64 years old female]

However, a few participants described symptoms like shortness of breath, dizziness, fatigue, joint pain, chest pain, *cough, fever*, loss of concentration, insomnia, loss of appetite, and depression.

*"I have information on the following signs and the symptoms of long COVID-19 like fatigue, cough, chest pain, and fever."* [35 years old male]

Most of the participants did not have information about the communicability of the virus during experiencing long COVID-19 symptoms. However, some participants perceived the virus as not transmissible, and other participants perceived the possibility of transmission from individuals with long COVID-19 symptoms.

*"I know that the COVID-19 virus cannot be transmitted after recovery because the virus is already cleared from the body. The symptoms come due to damage to the vital organs. And the symptom will continue until the damaged organ gets recovered."*[38 years old female]

*"I think individuals with long COVID-19 symptoms can transmit the virus. Even if the treatment can weaken its pathogenicity, the virus is still inside and is not cleared. So the possibility of transmitting the virus is still there."*[40 years old male]

Regarding the risk groups, most of the study participants were not aware of the risk groups. There is also a perception that COVID-19-vaccinated individuals are at risk for long COVID symptoms. However, a few participants considered females, individuals with comorbidities, and hospitalized individuals as the risk groups for long COVID-19 symptoms.

*"My friend has long COVID-19 symptoms but he perceived that these symptoms came due to the COVID-19 vaccine. Therefore, it is associated with the vaccine. No person complains about COVID-19."* [73 years old male]

*"I think individuals with older age and comorbidity such as diabetes; cancer and AIDS are at risk for long COVID-19 because of declined immunity."*[43 years old male]

On the contrary, some participants perceived non-hospitalized patients are a risk group due to getting less medical care when they do not admit to the hospital.

*“There is a difference between hospitalized and non-hospitalized individuals. Because none hospitalized individuals do not receive medical care. It is obvious that medication can reduce the risk of many symptoms.”* [43 years old male]

The majority of the respondents described that long COVID-19 symptoms will go off by themselves. However, they perceived symptoms will go off quickly through homemade care, lifestyle modification, and physical exercise.

*'Though the symptoms will go off by themselves, it is good to strengthen yourself through physical exercise, eating fresh foods, and keeping personal hygiene."*[40 years old male]

However, a few study participants perceived that long COVID-19 symptoms need medical care.

*“Persons with long COVID-19 symptoms should seek medical care as those symptoms may cause further complications.”* [40 years old male]

### Theme II: Experience and Effect of Long COVID Symptoms

The survivors experienced general, respiratory, cardiac, digestive, neurological, and other symptoms. These symptoms include rash, fatigue fever, cough, palpitations, shortness of breath, chest pain, abdominal pain, diarrhea, loss of concentration, loss of smell, sleep disorder, depression, and joint and muscle pain. These symptoms brought various physical and psychosocial effects for the participants.

Physical effects include inching, facial discoloration, unable to talk due to cough, forgetfulness, shaking hands, unable to perform daily activities, severe headache, and olfactory dysfunction. The psychosocial effect includes suicidal ideation and social isolation.

*"The long COVID-19 symptoms caused facial discoloration to me. I have serious inching thoughts my body likes street children having dermatitis."* [59 years old male]

*"I have serious muscle pain. I cannot protect myself from different incidents like dog bits and attacks from a mentally ill person. Even I cannot hold an umbrella.''* [73 years old male]

*"I had a serious headache for a long time. My head doesn't seem mine. Even it did not respond with anti-pain…. In addition, my neighbors greet me at a far distance [They are not happy to talk with me]. Even others are not voluntary to say hello [they perceive that the virus will be transmitted to them]. They isolate me from social events. I thought to commit suicide"* [43 years, male]

*“I have severe fatigue. I cannot go to church to pray and cannot receive "kidus kurban.”* [57 years old female]

*“I totally lost my memory. I cannot find files that I used to easily pick up on my computer before the illness. It highly affected my life and it persists up to now”* (40 years, male).

### Theme III: Survivor's care-seeking behavior for long COVID 19

Some survivors seek modern health care for severe symptoms such as muscle and joint pain, stomach pain, and chest pain. They visited to get a diagnostic evaluation, medicine, and different advice.

*"I used to visit my doctor. He gave me different medicines. He also advised me to take enough rest, and the foodstuffs I need to take. He also follows my progress with X-rays and different diagnostic evaluations at different times. This made me have an improvement."* [50 years old male]

Some survivors used to practice different spiritual care either as the only intervention or in combination with modern health care. The spiritual care practices include praying, holly water, and applying Eminent (  ).

*“In addition to modern care, I pray in the church. I used holy water and eminet* *(* *)*. *I massage my body with “Eminet”* *(* *)* *and baptized with holy water. I go to church on Saturday and Sunday to bring holy water and I drink the holy water. I put "Eminet” on my joint that pains me and massaged as well as with the holy water."* [70 years old female]

Some survivors practiced homemade care like applying butter on the head for headaches, avoiding caffeinated drinks, avoiding contact with laptop and mobile, drinkin*g* warm water for insomnia, use of garlic, and black cumin, taking hot drinks for chest pain, and eating fruits and salads for loss of appetite. In addition, survivors were doing physical exercise.

*“I asked my sister to apply butter to my head when I had a headache and get relief as soon as she applies it. I wash my head after three days to prevent a foul smell.”* [50 years old male]

*"I avoided beverages and other stimulants, contacts with the different screens [like laptop and mobile], and as much as possible I used to drink warm water, especially during the time of sleep."*[40 years old male]

*“Since the taste was not good for me, I was not eating. I used to eat fruits and salads. I used to take a lot of hot drinks like pourage, tea with ginger, garlic, and like that because I had chest pain.”* [38 years old male]

However, some survivors did not seek any intervention despite they were having severe symptoms.

*"I have serious muscle pain. I cannot hold an umbrella; I cannot protect myself from dogs or mentally ill people. Even I cannot hold my grandkid. However, I did not visit a health facility. "* [73 years old male]

## Discussion

This Phenomenological study explored the awareness about and care seeking for long COVID 19 symptoms among COVID survivors in Bahir Dar City in 2022. Twenty three participants, most of them were male, hospitalized, and not vaccinated for COVID-19. Awareness (Symptoms, risk groups, and communicability), Experience and effects of the symptoms, care practice, and advice were identified themes. Most of the COVID-19 survivors did not mention the common long COVID-19 symptoms.

In this study, a few participants described common long COVID-19 symptoms namely shortness of breath, dizziness, fatigue, joint pain, chest pain, cough, fever, loss of concentration, insomnia, loss of appetite, and depression. However, none of the participants did mention common long COVID-19 symptoms like palpitations, headache, pin or needle feeling, loss of smell, diarrhea, stomach pain, rash, and change in the menstrual cycle. This is consistent with studies conducted in the United Kingdom [11] in which many were unable to make sense of their suffering an experience intensified by the absence of medical knowledge or guidance. This might be due to the concept long COVID-19 is new, the absence of national long COVID-19 guidelines, and limited awareness creation mechanisms.

Regarding the awareness of the risk groups of long COVID symptoms, most of the study participants were unaware of risk groups. However, few participants described being female, comorbidities and hospitalized patients were risk groups for long COVID symptoms. This finding was in agreement with studies done in Spain [17–19].

Some participants perceived non-hospitalized patients are a risk group due to getting less medical care when they were not admitted to a hospital. This is consistent with studies done in Egypt [20].

Regarding the communicability of the virus during experiencing long COVID-19 symptoms, few participants described that the virus cannot transmit from person to person. This is also mentioned in the WHO guideline for long COVID symptoms [21]. Most of the participants did not have information about the communicability of the virus during experiencing long COVID-19 symptoms. On the contrary, a 40-year-old male participant perceived that the virus can be transmitted from person to person during experiencing long COVID-19 symptoms.

Participants with long COVID-19 experienced different symptoms respiratory and heart symptoms: cough, palpitations, shortness of breath, and chest pain. In addition, they reported neurologic symptoms including loss of concentration/focus, loss/change of smell, sleep disorder, and depression. These are also explained in the studies done in China [19, 22] and German [23].

Moreover, some of the participants also experienced rash, and joint and muscle pain. They feel pain in their calf, thigh, and ankle. It is also similar to the scope reviews done [24, 25].

In this study, participants had physical effects such as facial discoloration, shortness of breath, heart problems, forgetfulness, shaking hands, fatigue, cough, severe headache, and olfactory dysfunction. This is consistent with a study done in China [26].

In addition, the study participants explained the effect on the matter of psychosocial effects mainly stigmatized images that influenced the everyday life of long COVID patients through social isolation and social exclusion and instantly breaking down the system of social relationships because of considering long COVID-19 patients potentially infectious virus carriers. This finding is similar to other studies that showed patients face not only the threat of pain from the illness itself but also social stigma and discrimination [26, 27].

Participants who had long COVID symptoms were seeking modern care, spiritual care, traditional care, and lifestyle modifications. Those individuals who seek modern care have the opportunity to easily relieve the long COVID symptoms because they get the appropriate medical care early [23].

The traditional practices include applying butter on the head for headaches, avoiding caffeinated drinks and contact with a laptop and mobile and drinkin*g* warm water for insomnia, physical exercise, garlic, black cumin, taking hot drinks for chest pain. In addition, survivors with loss of appetite were eating fruits and salads. They practice spiritual care practices including praying, holy water and applying Eminent. There are similar studies done by the CDC (center for disease control and prevention) on the management of stress associated with long COVID conditions [22]. Moreover, studies done in the UK showed that they use traditional care for long COVID [28].

However, some survivors did not seek either modern care or other intervention despite they were having severe symptoms. This might be due to they consider the symptoms relief by their own.

## Conclusions

The result of this study revealed that participants have a significant deficit of awareness about the common symptoms, risk groups, and communicability of Long COVID in spite of the fact that they experienced the majority of the common symptoms of Long COVID. The participants experienced most of the common symptoms of Long COVID and the symptoms had differing degrees of severity ranging from simple ones to the most demanding physical and psychosocial effects including suicidal ideation. The experienced Long COVID symptoms highly affected not only the day-to-day routines but also the long-term physical and social functioning. To alleviate the problems they encountered, the participants took different measures including medical care, homemade remedies, spiritual solutions, and lifestyle modification.

## Recommendations

Based on the result of the study the following interventions are recommended. Health Professionals and research institutes should work on continuous awareness-creation activities for the community. Health facilities should provide diagnosis, care, and treatment services including psychosocial support, as well as counseling and follow-up activities for Long COVID-19 cases. The Ministry of Health and the regional health bureau should work with partners to avail a formally designed care and treatment for the care seekers. Researchers need to conduct longitudinal research to further the understanding of the disease.

## Data Availability

The datasets used and/or analyzed during this study are available from the corresponding author on reasonable request and approval from Bahir Dar University.
